# Application of Novel Lateral Tire Force Sensors to Vehicle Parameter Estimation of Electric Vehicles

**DOI:** 10.3390/s151128385

**Published:** 2015-11-11

**Authors:** Kanghyun Nam

**Affiliations:** School of Mechanical Engineering, Yeungnam University, 280 Daehak-ro, Gyeongsan 712-749, Korea; E-Mail: khnam@yu.ac.kr

**Keywords:** lateral tire force sensors, tire model, tire cornering stiffness estimation, lateral vehicle velocity estimation, ground vehicles

## Abstract

This article presents methods for estimating lateral vehicle velocity and tire cornering stiffness, which are key parameters in vehicle dynamics control, using lateral tire force measurements. Lateral tire forces acting on each tire are directly measured by load-sensing hub bearings that were invented and further developed by NSK Ltd. For estimating the lateral vehicle velocity, tire force models considering lateral load transfer effects are used, and a recursive least square algorithm is adapted to identify the lateral vehicle velocity as an unknown parameter. Using the estimated lateral vehicle velocity, tire cornering stiffness, which is an important tire parameter dominating the vehicle’s cornering responses, is estimated. For the practical implementation, the cornering stiffness estimation algorithm based on a simple bicycle model is developed and discussed. Finally, proposed estimation algorithms were evaluated using experimental test data.

## 1. Introduction

Vehicle dynamics control systems, such as yaw stability control, traction control and roll stability control systems, have developed for helping drivers maintain stability, maneuverability and safety [1,2]. In order to improve the control performances of those systems, a great deal of research on the estimation of vehicle states/parameters, required in dynamics control systems, has been done. In particular, studies on estimating the lateral vehicle velocity and tire cornering stiffness using various motion capture sensors have been carried out for enhancing the vehicle stability when cornering [1,3,4,5,6,7]. Real-time information on vehicle sideslip angle and tire cornering stiffness is critical in the yaw stability control system that is currently implemented in many ground vehicles to prevent spin-out and to match the vehicle’s cornering response to the driver’s intent. Unfortunately, these values are not easily obtained without using additional sensors. Due to the sensor cost issues, various types of estimators and observers have been employed in vehicle control systems [8,9,10]. A nonlinear observer using full state vehicle models was proposed to predict some instability vehicle behaviors when cornering [8]. Other estimation methods are for obtaining lateral vehicle velocity (or equivalently, vehicle sideslip angle) from vehicle dynamics and kinematics models or by conditionally switching between model-based estimation and kinematics-based estimation [1]. Since these methods are realized based on using vehicle dynamics models and tire models, estimation performances depend on the accuracy of the parameters used in the models. Considering that these parameters vary according to tire-road conditions and vehicle velocity, there exist limitations in estimation performances. For this reason, new estimation methods utilizing various kinds of sensors, such as Global Positioning System (GPS), multi-axis inertial sensors, tire force sensors, *etc.*, have been studied [11,12,13,14,15,16,17]. The GPS has been used for determining three-dimensional vehicle velocity measurements and used in estimation of vehicle sideslip angle and tire cornering stiffness without knowing the vehicle model [11,12,13]. However, estimation methods using GPS sensors require satellite visibility, which is periodically lost in urban and forested areas.

The vehicle state estimation approaches using tire force measurements have been studied for improving the accuracy of estimates and the robustness against parameter variations [1,4,14,15]. In [14], a method to evaluate the lateral vehicle states based on tire forces, directly measured by optical tire sensors, is proposed, and the Kalman filter estimator is presented. Since the measured tire forces are free from any bias from vehicle roll angle and road bank angle, it is also possible to obtain more reliable vehicle acceleration by using tire force measurements. In the literature [18,19], new load sensing hub bearing units, developed by SKF Corp., are introduced and used for enhancing the performances of the anti-lock braking system and the conventional electronic stability control system, respectively. In the author’s previous literature works [1,15], various methods for estimating the vehicle states, e.g., vehicle sideslip angle, roll angle, road friction coefficients, *etc.*, using lateral tire force sensors are proposed, and their effectiveness is discussed through comparative study and field test results. Force sensing hub bearings, developed by NSK Ltd., are used for obtaining tire force measurements.

This paper presents simple and practical methods for estimating the lateral vehicle velocity and tire cornering stiffness using lateral tire force measurements. The scheme of the lateral vehicle velocity estimator is an extension of Nam *et al.* [15]. Compared to the previous work [15], the effect of lateral load transfer across the vehicle is considered for enhancing estimation performance. In order to compensate for the adverse effect of load transfer, tire cornering stiffness is reformulated as a first-order polynomial with respect to the vertical tire force. Furthermore, an algorithm for identifying the tire cornering stiffness in real time is presented. The outline of this paper is as follows. In Section 2, lateral tire force sensors used in this work are introduced, and the sensing mechanisms are explained. Then, in Section 3, the vehicle and tire models are introduced. In Section 4, a procedure for the lateral vehicle velocity estimation and tire cornering stiffness is presented, and the applied estimation algorithms are introduced. In Section 5, experimental results are presented and discussed. This is then followed by concluding remarks in Section 6, which highlight the conclusion of this work and future works.

## 2. Lateral Tire Force Sensors

**Figure 1:**
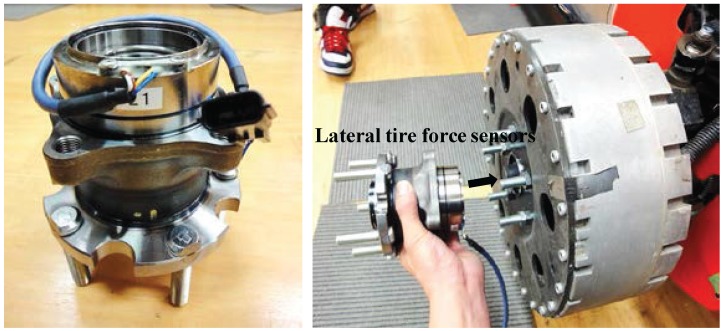
Real view of the lateral tire force sensor (*i.e.*, force-sensing hub bearing unit).

**Figure 2:**
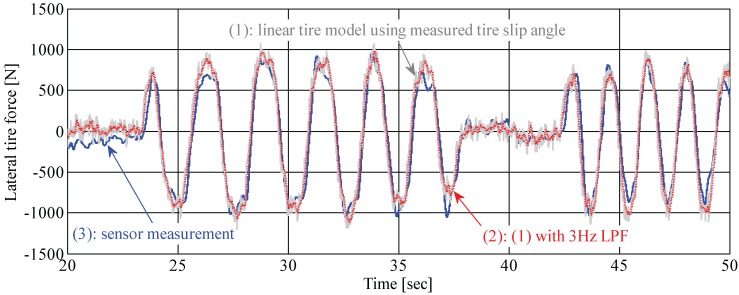
Lateral tire force measurement: (1) calculated force from a linear tire force model using measured tire slip angle and nominal tire cornering stiffness; (2) processed force of (1) using a low-pass filter with a cutoff-frequency of 3 Hz; (3) sensor measurement from a load-sensing hub bearing unit.

As the vehicle’s cornering responses are dominantly governed by the forces generated between the tire and the road, knowledge of individual tire forces in a lateral direction is very important when predicting and controlling vehicle cornering motion [20,21]. For this reason, several automotive component suppliers have tried to develop a novel device for directly measuring forces acting on tires. The NSK Ltd., a hub bearing manufacturer, invented a force-sensing hub bearing and is still making efforts for practical applications to commercial ground vehicles. In many commercial vehicles, wheel hub bearing units with built-in active anti-lock brake system sensors (*i.e.*, a wheel speed sensor) are equipped. Comparing force-sensing hub bearing units to conventional wheel hub units currently used in vehicles, force-sensing hub bearing units have almost the same weight and mechanical structure, except for rolling elements in a pair of rows. Furthermore, it is capable of being constructed at a low cost. The force-sensing principle is as follows: there are a couple of rolling elements (called inner and outer rings) in a hub bearing unit. Those are independently moved in the axial direction when lateral forces are acting on tires. A couple of magnetic-type encoders, which have N- and S-poles, are attached in an inner rolling element, and magnetic hall sensors are mounted on an outer rolling element. Two hall sensor outputs (*i.e.*, pulse signals with different phases) are used for calculating the lateral tire forces based on a pre-defined relation curve between the phase difference and tire force [22]. A lateral tire force sensor, *i.e.*, a force-sensing hub bearing unit, is illustrated in Figure 1, and a force measurement from the tire force sensor is represented in Figure 2, respectively. The result of Figure 2 was obtained from a field driving test with a smooth acceleration and a sine steering command.

(1) and (2) shown in Figure 2 are results for the indirect force measurement (*i.e.*, the calculated lateral tire force from a linear tire model using a measured tire slip angle and nominal tire cornering stiffness). (3) shown in Figure 2 is the measurement data of a force-sensing hub bearing unit. It should be noted that there exists a measurement error at low speeds (e.g., less than about 10 km/h vehicle speed), which can be seen in measurement data between 20 and 23 s. Since tire forces are calculated from the rotational signals, the signal-to-noise ratio is relatively low at a low speed.

## 3. Vehicle and Tire Models

In this section, the mathematical models for describing the dynamic motions of both a vehicle and tire are derived with details. Those models are used in establishing algorithms for estimating the lateral vehicle velocity and tire cornering stiffness.

### 3.1. Vehicle Modeling

**Figure 3:**
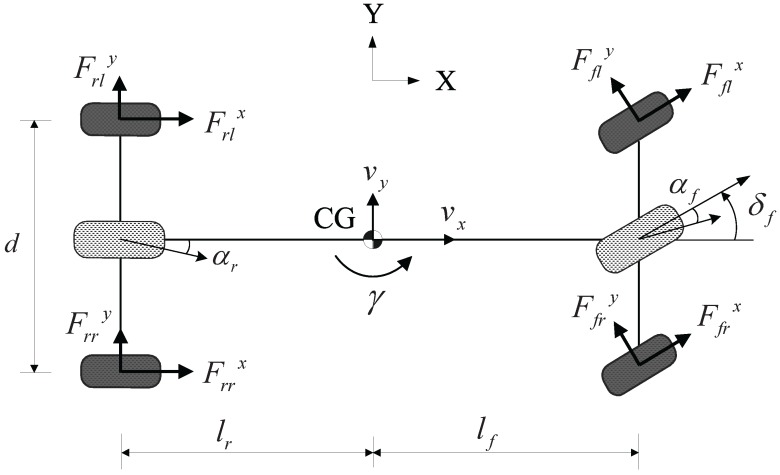
Schematic diagram of the vehicle model.

A schematic diagram of the vehicle model for describing lateral motion and yaw motion is shown in Figure 3. A body-fixed coordinate system with the origin at center of gravity (CG) is used to build the vehicle model.

The equations of motion governing the lateral and yaw dynamics of the ground vehicle with in-wheel motors (used in experiments) are given by:

(1) $$\begin{array}{cc} ma_{y} & =\left(F_{fl}^{x}+F_{fr}^{x}\right)\text{sin}\delta_{f}+\left(F_{fl}^{y}+F_{fr}^{y}\right)\text{cos}\delta_{f}+\left(F_{rl}^{y}+F_{rr}^{y}\right) \end{array}$$

(2) $$\begin{array}{cc} I_{z}\dot{\gamma } & =l_{f}\left(F_{fl}^{x}+F_{fr}^{x}\right)\text{sin}\delta_{f}+l_{f}\left(F_{fl}^{y}+F_{fr}^{y}\right)\text{cos}\delta_{f}-l_{r}\left(F_{rl}^{y}+F_{rr}^{y}\right)+M_{z} \end{array}$$

where *m* denotes the total mass of the vehicle, $a_{y}$ is the lateral acceleration at CG, *γ* is the yaw rate at CG, $l_{f}$ and $l_{r}$ are the distances from the vehicle CG to the front and rear axles, $I_{z}$ is the yaw moment of inertia and $\delta_{f}$ is the front steering angle. $F_{i}^{x}$ and $F_{i}^{y}$ are the longitudinal and lateral tire forces of the *i*-th wheel; *i* is fl, fr, rl and rr and represents the front left, front right, rear left, and rear right wheels, respectively; $M_{z}$ is the yawing moment generated by independent in-wheel motor control.

### 3.2. Lateral Tire Force Model

**Figure 4:**
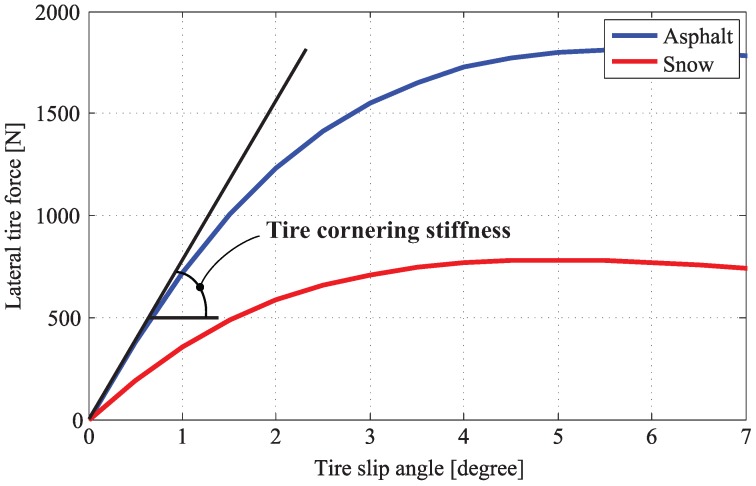
Relation between tire slip angle and lateral tire force.

Since cornering characteristics of a ground vehicle depend on lateral forces acting on tires, accurate tire models for calculating tire forces are required in vehicle dynamics controls. Over the past few decades, a great deal of research has been dedicated to the development of tire models [20], as a result of various tire models, such as Magic Formular, the Dugoff model and the Brush model, which have been proposed and applied to estimator and controller design. In this work, a linear tire model is used in estimator design for design simplicity. As shown in Figure 4, the relation between lateral tire force and tire slip angle is almost a straight line when the tire slip angle is small, and can be expressed as follows:

(3) $$\begin{array}{cc} F_{i}^{y}\;\; & =\;\;-C_{i}\text{tan}\left(\alpha_{i}\right) \end{array}$$

Here, $\alpha_{i}$ is the slip angle of the *i*-th tire and $C_{i}$ is the tire cornering stiffness, which is a sloped line, as shown in Figure 4.

**Figure 5:**
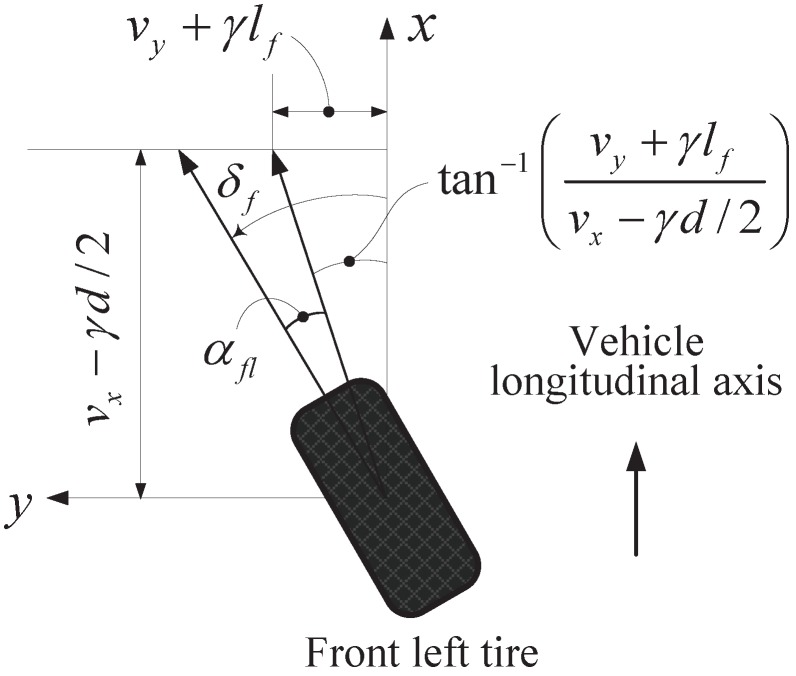
Calculation of the slip angle of a front left tire based on geometric derivation.

The individual tire slip angle $\alpha_{i}$ is calculated based on geometric derivation using wheel velocity vectors, as shown in Figure 5. If the velocities at the wheel ground contact points are known, the tire slip angles can be easily derived geometrically and are given by:

(4a) $$\begin{array}{cc} \alpha_{fl}\;\; & =\;\;-\delta_{f}+{\text{tan}}^{-1}\left(\frac{v_{y}+\gamma l_{f}}{v_{x}-\gamma d/2}\right) \end{array}$$

(4b) $$\begin{array}{cc} \alpha_{fr}\;\; & =\;\;-\delta_{f}+{\text{tan}}^{-1}\left(\frac{v_{y}+\gamma l_{f}}{v_{x}+\gamma d/2}\right) \end{array}$$

(4c) $$\begin{array}{cc} \alpha_{rl}\;\; & =\;\;{\text{tan}}^{-1}\left(\frac{v_{y}-\gamma l_{r}}{v_{x}-\gamma d/2}\right) \end{array}$$

(4d) $$\begin{array}{cc} \alpha_{rr}\;\; & =\;\;{\text{tan}}^{-1}\left(\frac{v_{y}-\gamma l_{r}}{v_{x}+\gamma d/2}\right) \end{array}$$

where $v_{x}$ is the longitudinal vehicle velocity, $v_{y}$ is the lateral vehicle velocity and *d* is the track width.

## 4. Vehicle State and Parameter Estimation

**Figure 6:**
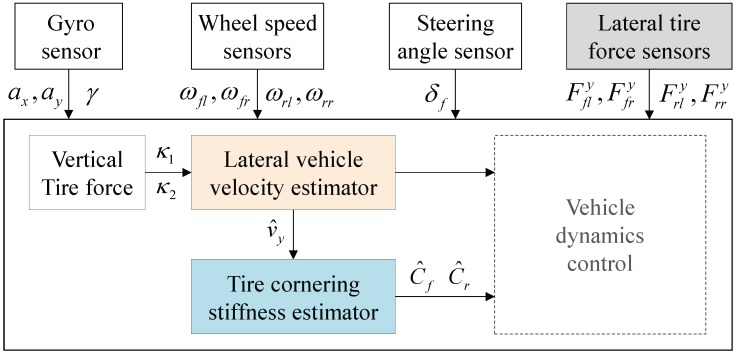
Illustration of lateral vehicle velocity and tire cornering stiffness estimators.

As mentioned in the Introduction, vehicle dynamics control systems require accurate estimates of vehicle states and parameters, such as lateral vehicle velocity, tire slip angles and tire cornering stiffness. For this reason, more accurate estimation methods using GPS or IMU were proposed, and their effectiveness has been widely discussed through a comparative study [12,13,16,17]. This section details a new method for estimating lateral vehicle velocity and tire cornering stiffness using lateral tire force measurements in conjunction with other available sensors (shown in Figure 6).

### 4.1. Estimation of Lateral Vehicle Velocity Using Tire Force Measurements

The methods of estimating lateral vehicle velocity using tire force measurements were presented in the author’s previous literature works [1,15]. A scheme of the lateral vehicle velocity estimator, proposed in this paper, is an extension of Nam *et al.* [15]. Compared to the previous work [15], an effect of lateral load transfer across the vehicle is considered for enhancing estimation performance.

From a linear tire force model Equation (3) and small angle approximation (*i.e.*, tan($\alpha_{i}$) ≈ $\alpha_{i}$), front left and right tire forces are expressed as follows:

(5) $$\begin{array}{cc} F_{fl}^{y} & =-C_{fl}\alpha_{fl}\;\approx \;-C_{fl}\left(\frac{v_{y}+\gamma l_{f}}{v_{x}-\gamma d/2}-\delta_{f}\right) \end{array}$$

(6) $$\begin{array}{cc} F_{fr}^{y} & =-C_{fr}\alpha_{fr}\;\approx \;-C_{fr}\left(\frac{v_{y}+\gamma l_{f}}{v_{x}+\gamma d/2}-\delta_{f}\right) \end{array}$$

**Figure 7:**
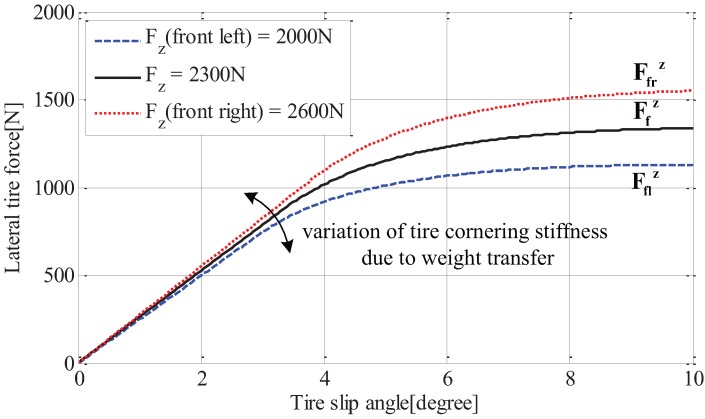
Characteristic curve between tire slip angle and lateral tire force with respect to load transfer.

It should be noted that tire cornering stiffnesses, $C_{fl}$ and $C_{fr}$, are the same during straight driving; however, the difference between those two values gradually increases as the lateral load transfer is becoming severe. The effect of tire vertical load on the tire cornering stiffness can be seen in Figure 7. That is, the tire cornering stiffness increases together with the vertical tire force.

To consider the effect of load transfer on tire cornering stiffness, we use the simplified tire cornering stiffness equation, which is represented by a first-order polynomial with respect to the vertical tire force, as shown as follows:

(7) $$\begin{array}{cc} C_{fl} & =\frac{2F_{fl}^{z}}{F_{fl}^{z}+F_{fr}^{z}}\cdot C_{f}=\kappa_{1}\cdot C_{f}\;\text{:Cornering}\;\text{stiffness}\;\text{for}\;\text{the}\;\text{front}\;\text{left}\;\text{tire} \end{array}$$

(8) $$\begin{array}{cc} C_{fr} & =\frac{2F_{fr}^{z}}{F_{fl}^{z}+F_{fr}^{z}}\cdot C_{f}=\kappa_{2}\cdot C_{f}\;\text{:Cornering}\;\text{stiffness}\;\text{for}\;\text{the}\;\text{front}\;\text{right}\;\text{tire} \end{array}$$

where $C_{f}$ is the average value for front cornering stiffnesses, $F_{fl}^{z}$ and $F_{fr}^{z}$ are the vertical forces acting on front left and right tires, respectively, which are calculated from the following equations (it is noted that lateral load transfer caused by body roll is not considered, because the load transfer by lateral acceleration is dominant),

(9a) $$\begin{array}{ccc} F_{fl}^{z}\; & =\;mg\left[\frac{l_{r}}{2l}-\frac{a_{x}}{g}\frac{h_{\text{CG}}}{2l}-\frac{a_{y}}{g}\frac{l_{r}h_{\text{CG}}}{dl}\right] & \;\text{:Front}\;\text{left}\;\;\; \end{array}$$

(9b) $$\begin{array}{ccc} F_{fr}^{z}\; & =\;mg\left[\frac{l_{r}}{2l}-\frac{a_{x}}{g}\frac{h_{\text{CG}}}{2l}+\frac{a_{y}}{g}\frac{l_{r}h_{\text{CG}}}{dl}\right] & \;\text{:Front}\;\text{right.} \end{array}$$

From Equations (7) and (8), lateral tire force equations, *i.e.*, both Equations (5) and (6), can be represented as:

(10) $$\begin{array}{cc} F_{fl}^{y} & =-C_{fl}\alpha_{fl}\;\approx \;-\kappa_{1}C_{f}\left(\frac{v_{y}+\gamma l_{f}}{v_{x}-\gamma d/2}-\delta_{f}\right) \end{array}$$

(11) $$\begin{array}{cc} F_{fr}^{y} & =-C_{fr}\alpha_{fr}\;\approx \;-\kappa_{2}C_{f}\left(\frac{v_{y}+\gamma l_{f}}{v_{x}+\gamma d/2}-\delta_{f}\right) \end{array}$$

By dividing Equation (10) by Equation (11), the lateral vehicle velocity can be derived as a form of regressor model.

The estimated parameter θ(t), input regression $\varphi^{T}\left(t\right)$ and measured output y(t) can be given as:

(12a) $$\begin{array}{cc} \theta (t)\; & =\;v_{y} \end{array}$$

(12b) φT(t)=(κ1−1Ffly(vx+γd/2−(κ2−1Ffry(vx−γd/2

(12c) y(t)=γlf(κ1−1Ffly(vx+γd/2−(κ2−1Ffry(vx−γd/2−δf(κ1−1Ffly−κ2−1Ffry)

where $\kappa_{1}$ and $\kappa_{2}$ are calculated by using calculated vertical tire forces, and a recursive least square algorithm (RLS) is also used to estimate the lateral vehicle velocity.

The recursive process of the RLS algorithm is described as [23]:

(13a) $$\begin{array}{cc} \hat{\theta }\left(t\right)\; & =\;\hat{\theta }\left(t-1\right)+K\left(t\right)\left(y\left(t\right)-\varphi^{T}\left(t\right)\hat{\theta }\left(t-1\right)\right) \end{array}$$

(13b) $$\begin{array}{cc} K(t)\; & =\;P\left(t-1\right)\varphi \left(t\right){\left[\lambda I+\varphi^{T}\left(t\right)P\left(t-1\right)\varphi \left(t\right)\right]}^{-1} \end{array}$$

(13c) $$\begin{array}{cc} P(t)\; & =\;\lambda^{-1}\left[I-K\left(t\right)\varphi^{T}\left(t\right)\right]P\left(t-1\right) \end{array}$$

where *I* is the identity matrix and K(t) and P(t) are the Kalman gain and covariance matrices.

In order to cope with time-varying properties in a vehicle system, the weighted least squares criterion is handled by putting less weight on older measurements. Therefore, the weighting function is set to [23]:

(14) $$\begin{array}{cc} \Gamma (t,k) & =\lambda^{t-k};\;i.e.,\;\lambda \left(t\right)\equiv \lambda \end{array}$$

where the choice of forgetting profile Γ(t,k) is conceptually simple. It is common to select it so that the least square criterion weighting essentially contains those measurements that are relevant for the current properties of the system [23]. For a system that changes gradually and in a “stationary manner”, the most common choice is to take a constant forgetting factor, such as Equation (14). The forgetting factor *λ* is always chosen to be a positive constant slightly smaller than one, so that $\Gamma \left(t,k\right)=e^{(t-k)\log\lambda }\approx e^{-(t-k)(1-\lambda )}$. This means that measurements that are older than $T_{0}=1/\left(1-\lambda \right)$ samples are included in the criterion with a weight that is $e^{-1}\approx 36\%$ of that of the most recent measurements. It is called a “memory time constant” of the criterion and is represented as:

(15) $$\begin{array}{cc} T_{0} & ={\left(1-\lambda \right)}^{-1} \end{array}$$

If the system remains approximately constant over $T_{0}$ samples, a suitable choice of *λ* can then be made from Equation (15). Typical choices of *λ* are in the range between 0.95 and 0.999. The smaller *λ* is, the less weight is assigned to the older data; that is, the past data are forgotten faster. In this work, *λ* around 0.995 was selected to make a reasonable trade-off between tracking ability and noise sensitivity.

### 4.2. Estimation of Tire Cornering Stiffness

In this section, a real-time algorithm for estimating tire cornering stiffness is presented. A single track vehicle model, also called the bicycle model, is used to build the estimator. By simplifying the differential Equations (1) and (2), linear dynamic equations describing the lateral and yaw motions for the bicycle model are obtained as follows:

(16) $$\begin{array}{cc} ma_{y} & =F_{f}^{y}+F_{r}^{y} \end{array}$$

(17) $$\begin{array}{cc} I_{z}\dot{\gamma } & =l_{f}F_{f}^{y}-l_{r}F_{r}^{y}+M_{z} \end{array}$$

where $F_{f}^{y}\left(=F_{fl}^{y}+F_{fr}^{y}\right)$ and $F_{r}^{y}\left(=F_{rl}^{y}+F_{rr}^{y}\right)$ are the front and rear lateral tire forces, which can be approximated as follows:

(18) $$\begin{array}{cc} F_{f}^{y} & =-2C_{f}\alpha_{f}=-2C_{f}\left(\frac{v_{y}+\gamma l_{f}}{v_{x}}-\delta_{f}\right) \end{array}$$

(19) $$\begin{array}{cc} F_{r}^{y} & =-2C_{r}\alpha_{r}=-2C_{r}\left(\frac{v_{y}-\gamma l_{r}}{v_{x}}\right) \end{array}$$

Here, it is noted that lateral tire forces, $F_{f}^{y}$, $F_{r}^{y}$, can directly be measured using force-sensing hub bearing units; *γ* is also measured by a gyro sensor; the longitudinal vehicle velocity $v_{x}$ can be calculated by averaging the non-driven wheels’ velocities; the front steering angle $\delta_{f}$ is measured by a steering angle sensor; and the lateral vehicle velocity $v_{y}$ is replaced with the value ${\hat{v}}_{y}$, *i.e.*, estimated lateral vehicle velocity in Section 4.1.

In a similar manner, tire cornering stiffnesses ($C_{f}$, $C_{r}$ in Equations (18) and (19)) are identified using the following regression model, which is built from Equations (16)–(19).

(20) $$\begin{array}{cc} y(t)\; & =\;\varphi^{T}\left(t\right)\theta \left(t\right) \end{array}$$

where the parameter to be identified θ(t) and measured output y(t) are chosen as:

(21) $$\begin{array}{cc} \theta \left(t\right)=\left[\begin{array}{c} C_{f}\\ C_{r} \end{array}\right],\;\;\;y\left(t\right) & ={\left[\begin{array}{c} ma_{y},\;I_{z}\dot{\gamma }-M_{z} \end{array}\right]}^{T} \end{array}$$

Furthermore, regression vector $\varphi^{T}\left(t\right)$ is given by:

(22) $$\begin{array}{cc} \varphi^{T}\left(t\right) & =\left[\begin{array}{cc} -2{\hat{\alpha }}_{f} & -2{\hat{\alpha }}_{r}\\ -2l_{f}{\hat{\alpha }}_{f} & 2l_{r}{\hat{\alpha }}_{r} \end{array}\right] \end{array}$$

where $\hat{\alpha_{f}}$ and $\hat{\alpha_{r}}$ are:

(23) $$\begin{array}{c} \hat{\alpha_{f}}=\left(\frac{{\hat{v}}_{y}+\gamma l_{f}}{v_{x}}-\delta_{f}\right)\;\;\text{and}:\;\;\hat{\alpha_{r}}=\left(\frac{{\hat{v}}_{y}-\gamma l_{r}}{v_{x}}\right) \end{array}$$

Tyre cornering stiffness in the aforementioned equations is also estimated by making use of the recursive least square (RLS) algorithm, i.e., Equations (13a)–(13c).

## 5. Experimental Verification

**Figure 8:**
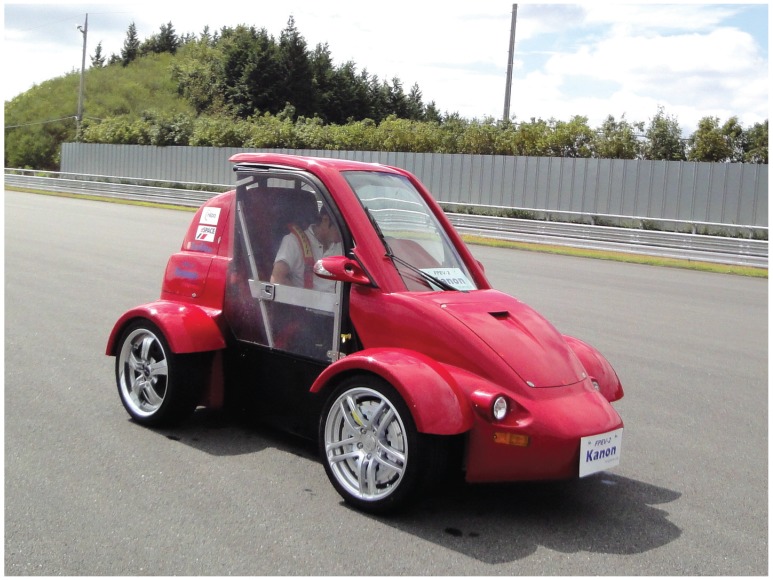
Experimental in-wheel motor-driven electric vehicle.

This section demonstrates the proposed estimation algorithms by implementing them with the experimental vehicle shown in Figure 8. The field tests consisting of $\pm 60^{\circ }$ steering maneuvers at a constant speed were performed to validate the lateral vehicle velocity and tire cornering stiffness estimation algorithms.

### 5.1. Experimental Setup

**Figure 9:**
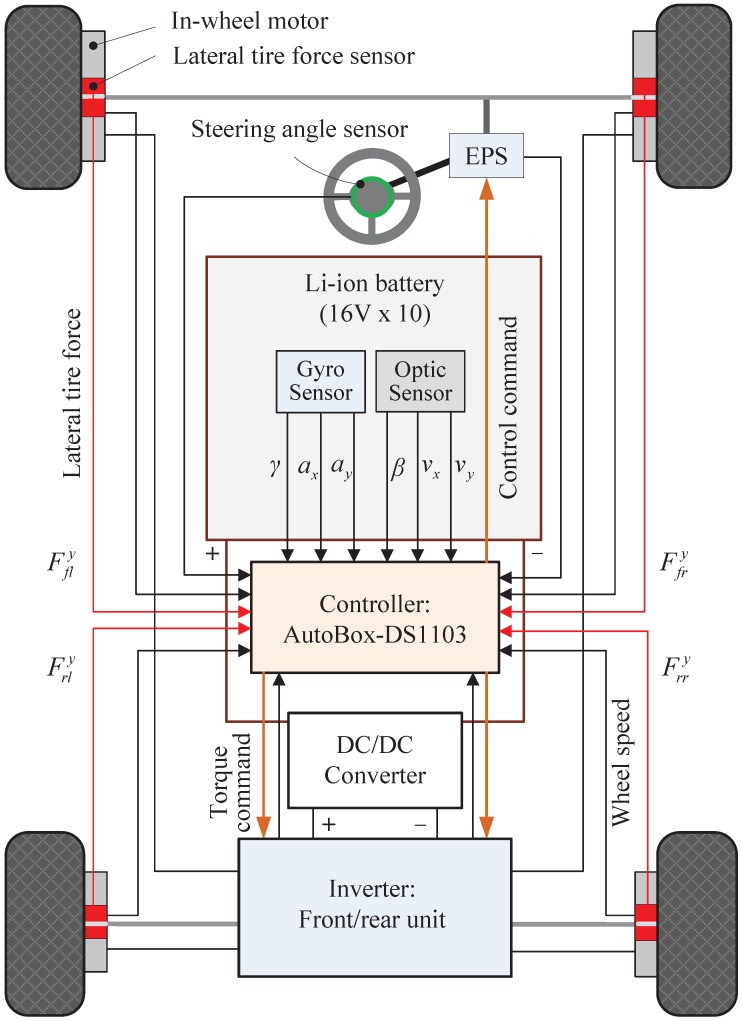
Description of the system interface of the experimental electric vehicle.

The experimental vehicle used in this work was developed by the Hori/Fujimoto research team at The University of Tokyo and has the following features (please see Figure 9) [1,15].

- In-wheel motors are attached in each wheel, and these are independently controlled by specially-designed motor control units, including inverters and digital signal processors.
- Novel sensors for sensing lateral tire forces are used for practical applications to vehicle dynamics control systems.
- Other sensors, which are now available in commercial ground vehicles, are also attached to the experimental vehicle, e.g., a steering angle sensor, a gyro sensor and wheel speed sensors.
- A non-contact optical sensor, Correvit (Corrsys-Datron), is used for accurate measurements of sideslip angle, lateral vehicle velocity and longitudinal vehicle velocity. That is only used as a reference for validating the proposed estimation algorithms.
- The dSPACE AutoBox (DS1103), which consists of a power PC 750GX controller board running at 933 MHz, 16-channel A/D converter and 8-channel D/A converter, was used for both real-time data acquisition and implementation of the estimation algorithms.
- The specifications for the experimental electric vehicle are listed in Table 1.

**Table 1:** Specifications of the experimental electric vehicle.

Total mass (m)	875 kg
Distance between CG and front axle ($l_{f}$)	1.013 m
Distance between CG and rear axle ($l_{r}$)	0.702 m
Track width (*d*)	1.3 m
Height of CG ($h_{CG}$)	0.51 m
Yaw moment of inertia ($I_{z}$)	617 kg· m${}^{2}$

### 5.2. Experimental Results

**Figure 10:**
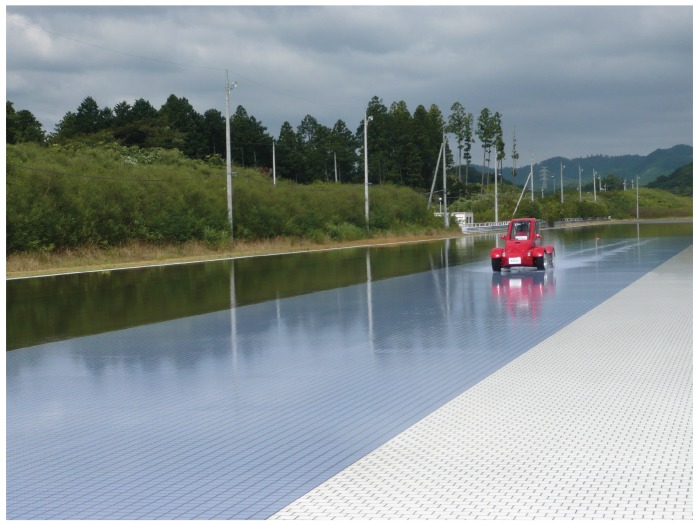
Field test on low-*μ* ground (μ≃0.3).

Field tests on a proving ground (as shown in Figure 10) were carried out for evaluating the estimation performances. The same steering maneuver has been done on dry asphalt (*i.e.*, μ≃0.9) and a slippery road (*i.e.*, μ≃0.3), respectively.

**Figure 11:**
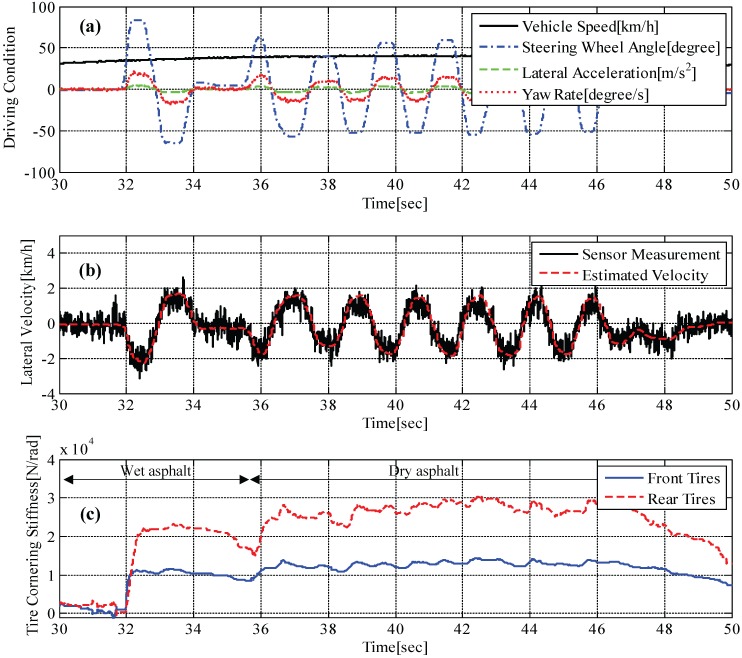
Test result on a high-*μ* road: (**a**) maneuvering conditions, e.g., vehicle speed, steering angle, measured lateral acceleration and measured yaw rate; (**b**) measured lateral vehicle velocity and estimated lateral vehicle velocity; (**c**) estimated tire cornering stiffness.

Figure 11 illustrates the results for lateral vehicle velocity estimation and tire cornering stiffness estimation. The lateral vehicle velocity was estimated by solving Equations (13a)–(13c) at each time step (*i.e.*, 0.001 s) using available sensor measurements, including lateral tire forces, as shown in Figure 6. Figure 11b shows that the estimated velocity (*i.e.*, dotted line) is well matched with the measured velocity (*i.e.*, solid line) by an optical sensor. In a similar manner, tire cornering stiffness was estimated, and the result is shown in Figure 11c. Since the vehicle ran on wet asphalt for around 35.5 s and after that ran on dry asphalt at a constant speed, slightly different estimates are examined. From the estimation results between 36 s and 46 s, we can confirm that the estimated front tire cornering stiffness approaches the calculated value shown in Figure 13. We could also confirm that the tire cornering stiffness is higher on dry asphalt than on wet asphalt, as expected.

**Figure 12:**
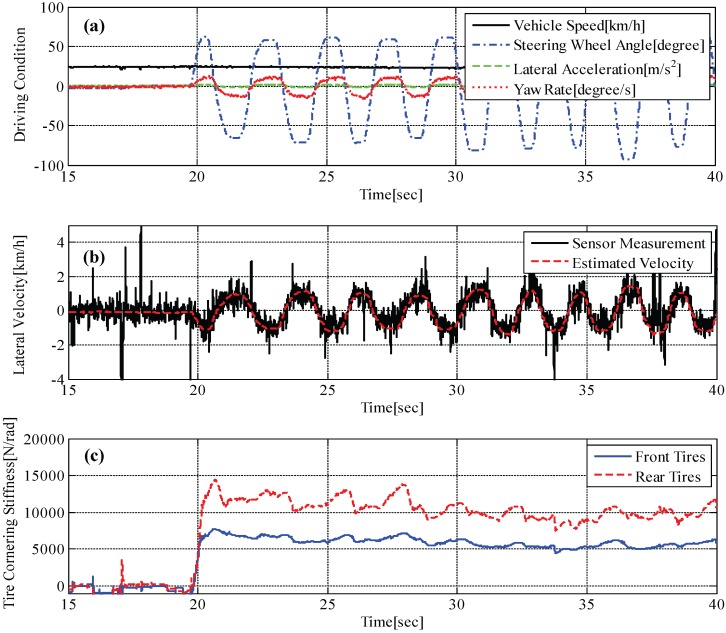
Test result on a low-*μ* road: (**a**) maneuvering conditions, e.g., vehicle speed, steering angle, measured lateral acceleration and measured yaw rate; (**b**) measured lateral vehicle velocity and estimated lateral vehicle velocity; (**c**) estimated tire cornering stiffness.

With similar driving maneuvers, experimental tests on a slippery road were performed, and the results are shown in Figure 12. It is confirmed that the proposed estimator provides very accurate estimation without any noticeable phase lag, as shown in Figure 12b. In addition, estimation values for front and rear tire cornering stiffnesses are close to the values of the calculated tire cornering stiffnesses using Equations (18) and (19). Since we cannot directly measure the tire cornering stiffness using sensors, the calculated tire cornering stiffnesses using Equations (18) and (19) are used as a reference for validating the estimation algorithms. It should be noted that the field tests were carried out at a relatively low speed (*i.e.*, around $v_{x}=40$ km/h) due to limited driving conditions, where the amount of lateral tire forces are small. This causes a low signal-to-noise ratio, which deteriorates the estimation performances. It is expected that the proposed estimators can provide improved estimates of lateral vehicle velocity and tire cornering stiffness if field tests are performed at a higher vehicle speed.

**Table 2:** Calculated values for tire cornering stiffness.

Road Condition	$C_{f}$ (N/rad)	$C_{r}$ (N/rad)
Dry asphalt (μ≃0.9)	12500	29200
Slippery road (μ≃0.3)	5900	11400

**Figure 13:**
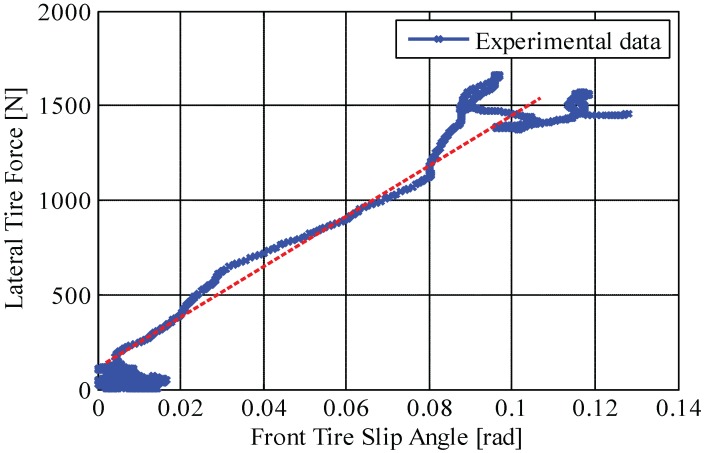
Front tire cornering stiffness on a high-*μ* road (μ≃0.9): relationship curve between measured lateral tire force and measured tire slip angle.

**Figure 14:**
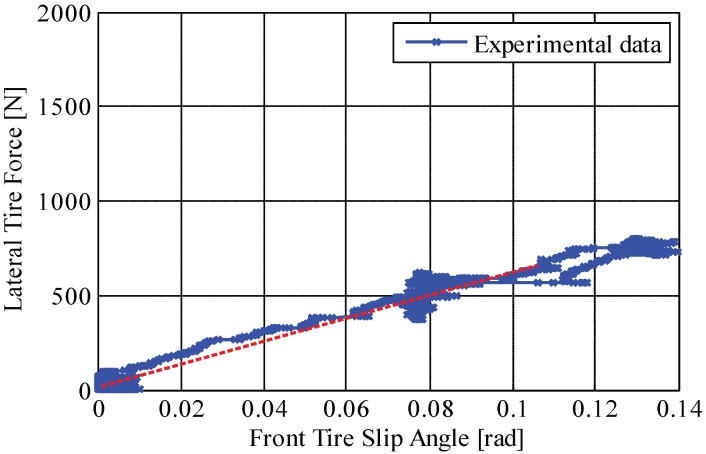
Front tire cornering stiffness on a low-*μ* road (μ≃0.3): relationship curve between measured lateral tire force and measured tire slip angle.

Tire cornering stiffnesses calculated by Equations (18) and (19) are listed in Table 2. The values are obtained from the test results shown in Figure 13 and Figure 14, illustrating the relation between measured lateral tire forces and measured tire slip angle. As mentioned, the gradient (*i.e.*, red dotted line) represents tire cornering stiffness.

## 6. Conclusions

This paper proposes methods for estimating lateral vehicle velocity and tire cornering stiffness using lateral tire force measurements. The feasibility of applying lateral tire force sensors to ground vehicles was validated through the evaluation of field test results. For estimating the lateral vehicle velocity, tire force models, considering the effects of load transfer on tire cornering stiffness, are used to build the recursive least square algorithm. In addition, tire cornering stiffnesses, which are critical factors deciding vehicle cornering responses, are estimated by using the estimated lateral vehicle velocity and the bicycle model. Both estimation algorithms are implemented on an experimental electric vehicle, and estimation results are obtained from field tests. Through test results, it is confirmed that novel tire force sensors can be applied to vehicle dynamics control systems for providing more accurate estimates without additional cost. In future works, a variety of control and estimation methods utilizing lateral tire force sensors are proposed and discussed.
